# Validation study to determine the accuracy of central blood pressure measurement using the SphygmoCor XCEL cuff device in patients with severe aortic stenosis undergoing transcatheter aortic valve replacement

**DOI:** 10.1111/jch.14245

**Published:** 2021-05-04

**Authors:** Jose M. De la Torre Hernández, Gabriela Veiga Fernandez, Jonathan Brown, Fermin Sainz Laso, Dae-Hyun Lee, Victor Fradejas, Tamara Garcia Camarero, Sammy Elmariah, Ignacio Inglessis, Javier Zueco, Jose A. Vazquez de Prada, Eyal Ben-Assa, Elazer R. Edelman

**Affiliations:** 1 Cardiology Division, Hospital Universitario Marques de Valdecilla, IDIVAL, Santander, Spain; 2 Institute for Medical Engineering and Science, Massachusetts Institute of Technology, Cambridge, MA, USA; 3 Cardiovascular Division, Brigham and Women’s Hospital, Harvard Medical School, Boston, MA, USA; 4 Cardiology Division, Department of Medicine, Massachusetts General Hospital, Harvard Medical School, Boston, MA, USA; 5 Department of Cardiology, Medical School, University of Cantabria, Santander, Spain; 6 Cardiology Division, Assuta Ashdod University Hospital, Ben Gurion University, Ashdod, Israel

## Abstract

Central aortic blood pressure could be helpful in the evaluation of patients with aortic stenosis (AS). The SphygmoCor XCEL device estimates central blood pressure (BP) measurement with its easy-to-use, operator-independent procedure. However, this device has not been properly validated against invasive measurement in patients with severe AS. We evaluated the relationship between cuff-brachial BP, transfer function- estimated and invasively measured central aortic pressure in patients with severe AS before and after transcatheter aortic valve replacement (TAVR). Agreement between techniques was analyzed and, according to the ARTERY Society recommendations, the minimum acceptable error was a mean difference ± SD ≤5 ± ≤8 mm Hg. A total of 94 patients with AS undergoing TAVR had simultaneous non-invasive and invasive measurements of central BP before and after the procedure. Before TAVR central systolic BP was in average slightly underestimated, though with wide variability, when using the default calibration of brachial-cuff SBP (mean difference ± SD, −3 ± 15 mm Hg), and after TAVR the degree of underestimation increased (mean difference ± SD, −9 ± 13 mm Hg). The agreement tended to improve for those patients with low aor- tic gradient stenosis compared to those with high gradient at baseline (mean dif- ference ± SD, −2 ± 11 mm Hg vs. −4 ± 17, respectively, *p* = .3). The cuff-brachial systolic BP yielded numerically lower degree of agreement and weaker correlation with invasive measurements than SphygmoCor XCEL. In patients with severe AS the SphygmoCor XCEL cuff device, despite showing strong correlation, does not meet the ARTERY Society accuracy criteria for non-invasive measurement of central SBP.

## 1 | INTRODUCTION

Calcific aortic stenosis (AS) is the most prevalent heart valve disorder in developed countries. Today, we still focus on symptoms in determining the timing of intervention, except for those showing depressed left ventricular (LV) systolic function. Yet, we are still guided by subjective reporting of symptoms leading to the potential of missing the optimal time for intervention.^1^ Aortic valve stenosis should be understood as a complex, multifaceted, systemic disease that is not solely limited to the aortic valve but also includes reduced arterial compliance as well as alterations of LV geometry and function.^2–4^

Several physiological studies, using either invasive or noninvasive based methodologies, have demonstrated the importance of the interaction between the aortic, ventricular and vascular components in AS patients and established its implication on clinical improvement after transcatheter aortic valve replacement (TAVR).^5,6^

Nonetheless, there is no well-validated quantitative framework integrating the interaction and coupling of left ventricle, aortic valve, and vasculature, which could guide not only who and when to intervene but as to how to optimize treatment following intervention. In order to achieve this landmark milestone, it is key to have reliable methods to estimate the hemodynamic conditions of the patient with AS in a non- invasive way.^6^ In this regard, the central aortic pressure is an essential parameter to be used in these approaches. There are several commercially available systems purporting to estimate central pressure, and these may be useful for refining risk stratification related to hypertension.^7^ In 2017, the ARTERY Society published recommendations on ap propriate validation protocols for central BP devices, and this included a requirement for invasive central BP being the reference.^8^

Estimation of differences between central and brachial pressures is also of particular practical relevance in AS, allowing a precise assessment of hypertension and vascular stiffness in these patients which contributes independently to left ventricular hypertrophy, systolic and diastolic dysfunction, and even prognosis.^9^ In addition, central pressure provides a more accurate measure of the summa- tive effects of systemic arterial hypertension upon the LV and hence of the LV wall stress.

We sought a non-invasive method that derives central pressure from peripheral measurements, using the SphygmoCor XCEL device (AtCor Medical). This device uses a generalized transfer function (GTF) and standard blood pressure measurements to derive the central aortic pressure waveform from the brachial volume displacement waveform. This method has been evaluated in two appropriately powered validation studies in which both the device underestimated central SBP but to a different extent, passing the criteria established by the ARTERY Society protocols^8^ in one study,^10^ but not in the other.^11^

In patients with AS the SphygmoCor XCEL device has not been evaluated for validation and this is a pertinent aim. The original transfer function was not validated in the setting of AS, and accordingly, this system is not labeled by FDA for the use in patients with AS. Furthermore, due to the narrowing of the valve, Venturi effect, and drop in pressure create a very different flow environment from what other non-AS studies have examined with this device. Moreover, the dynamics in agreement before and after aortic valve replacement have not been evaluated yet. A different non-invasive method for estimation of central pressures based on radial artery tonometry was applied in patients with AS showing a suboptimal agreement, though the number of patients included (*n* = 14) is an important limitation. ^12^

We conducted a validation study aimed to determine the accuracy of the SphygmoCor XCEL device compared with invasively measured central aortic BP in patients with severe AS undergoing TAVR.

## 2 | METHODS

Prospective international registry conducted at two institutions in Spain (HUMV, Santander) and the United States of America (MGH, Boston, MA), during 2019–2020.

Patients scheduled to undergo TAVR were included if meeting the following criteria:

Inclusion criteria:

1. Diagnosis of severe and symptomatic AS;
2. indication for TAVR established by the institutional Heart Team;
3. undergoing a TAVR procedure through a femoral artery access. Exclusion criteria: (1) Patients not consenting; (2) patients showing some degree of cognitive impairment that prevented them from properly understanding the investigational procedures.

Patients enrolled in the study but suffering any kind of major complication during the TAVR procedure that precluded the recording of pressure tracings were excluded from the analysis. Methods were undertaken according to the ARTERY Society recommendations, and a minimum acceptable error was ≤5 mm Hg for mean difference and ≤8 mm Hg for SD. ^8^

The study was approved by the corresponding Institutional Review Board and all participating patients signed the informed consent after proper explanation of the investigational procedures. Database was completely anonymized.

### 2.1 | TAVR procedure

All procedures were performed under general anesthesia, monitored by an anesthesiologist in the appropriate setting of a Cath Lab dedicated to structural heart interventions. The interventional team consisted of two interventional cardiologists with a large experience in TAVR procedures, an interventional cardiology fellow in charge of the investigational procedures, an echocardiographist specialized in structural heart interventions, and two nurses with their corresponding circulating assistants. In addition, a vascular surgeon was present in the Cath Lab in those cases not deemed appropriate for percutaneous access. The procedure was done following the adequate standard techniques.

### 2.2 | Non-invasive central pressure measurements

The non-invasive measurements were taken at two different time points during the TAVR procedure. (1) Intra-procedure pre-TAVR, performed after full sedation of the patient and insertion of all catheters required, in stable hemodynamic conditions and before any kind of intervention over the aortic valve (either balloon dilatation or prosthetic valve implantation). (2) Intra-procedure post-TAVR, after pros- thetic valve implantation and stabilization of hemodynamic conditions, excluding any kind of complications and not showing pacemaker dependence. This last measurement was generally taken 10–15 min after valve implantation and just before large-bore sheath removal.

Non-invasive central pressure was captured with the SphygmoCor XCEL device. Pulse wave contours were formed by averaging several heartbeats. For those patients with a history of arrhythmias, care was taken during measurement to ensure that the waveforms that were chosen were best representing.

A dedicated BP cuff connected to the XCEL device was placed on a subject’s arm, and an automated sequence measured pulse wave contours in the following manner. Three consecutive BP mea- surements were taken, and the average of the final two was used for pulse waveform calibration. The cuff was then re-inflated to a sub-diastolic pressure, and the pulse wave contours of 10 heart- beats were recorded and averaged. This averaged waveform was digitized and saved for offline processing and analysis. Three repeated measurements were performed and averaged to calculate the non-invasively measured central systolic blood pressure (SBP) and central pulse pressure (PP) values. All measurements were taken by specifically trained personal with large experience in the use of this system in the setting of the hypertension clinic.

### 2.3 | Invasive aortic pressure measurements

Simultaneously with the non-invasive recording of the central pressure, pressure in the ascending aorta was measured invasively with a 5 or 6 Fr pigtail catheter attached to a fluid-filled manometer sys- tem. The system manifold was maintained on the catheter table at a height equivalent of the heart, zero calibrated to atmosphere before catheterization and the system was flushed before continuous acquisition of invasive central BP waveforms. All pressure waveform signals were acquired at a sample rate of 1000 Hz via an analog-to- digital converter and recorded. The catheter was inserted through a femoral 6 Fr introducer sheath (contralateral to the femoral access for TAVR) with its tip steadily positioned in the mid-portion of the ascending aorta, at least 3 cm over the cusps of the aortic valve. Invasive aortic pressure recordings were taken at two different intra-procedural moments, pre- and post-TAVR, simultaneously with the non-invasive ones. The average of 20 cardiac cycles was used to render the final pressure measurements. The 5 s period of invasive central BP waveforms corresponding directly with the time of Xcel cuff waveform capture were ensemble-averaged for analysis.

**FIGURE 1:**
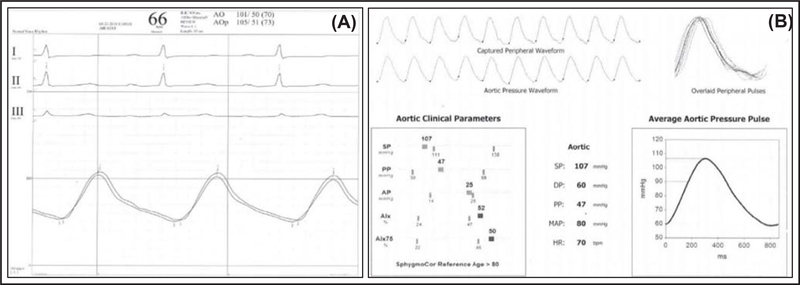
Blood pressure recordings with invasive and non-invasive approaches. (A) Curves for aortic and femoral pressures recorded during TAVR procedure through the fluid-filled catheter. (B) Curves for central and brachial pressures obtained with SphygmoCor XCEL simultaneously with the invasive recordings

The Figure 1 shows the pressure tracings of the two methods used for simultaneous BP measurements, the invasive aortic and femoral pressures recorded in the polygraph during the TAVR pro- cedure, and the central and brachial pressures curves captured non- invasively depicted in the SphygmoCor report.

### 2.4 | Echocardiographic procedures

All patients underwent a systematic protocol-specific transthoracic echocardiography examination just before entering the Cath Lab and after TAVR before discharge. A systematic protocol-specific transesophageal echocardiography was done during the TAVR procedure, before and after prosthetic valve implantation. The intra- procedural transesophageal echocardiographic measurements were taken simultaneously with the invasive and non-invasive central pressure measurements. Additional echocardiographic studies could be done as clinically indicated during the post-TAVR period. A complete set of data were collected addressing morphological and functional aspects of the aortic valve, the left ventricle, and the ascending aorta.

### 2.5 | Statistics

The characteristics of patients are shown as mean (SD) for continuous variables or median (interquartile range) for those not following normal distribution. Distribution was assessed for each variable with the Kolmogorov-Smirnov test. Scatter diagrams and regression lines were conducted for the overall cohort and for the considered sub- groups. The Bland-Altman plots or difference plots were obtained as a graphical method to determine magnitude and direction of any systematic bias. Pearson’s coefficient of correlation was used to examine the association between measurements obtained using the different techniques. A multiple regression analysis was conducted to identify independent predictors of bias between invasive and non-invasive SBP. Two-tailed *p* values <.05 were considered to be statistically significant. All data were analyzed using SPSS for windows version 20 and MedCalc version 15.

## 3 | RESULTS

This study included a total of 99 patients that met all inclusion and exclusion criteria. Among these, in five patients no adequate measurements could be obtained because of intra-procedural compli- cations ^3^ or technical issues.^2^ Baseline characteristics of the study population are shown in Table 1. Mean age was 81 ± 6.7 years with 47% of women and the vast majority (83%) had hypertension diagnosed and treated before the TAVR procedure.

All TAVR procedures were performed through a transfemoral approach with a balloon-expandable TAVR system with a procedural successful result in all of them. During hospital admission, one patient suffered a stroke and one patient had a myocardial infarction. Vascular and bleeding complication rates according to the VARC-2 and BARC consensus definitions are shown in the Table 2.

The baseline echocardiographic parameters showed mean transaortic gradient of 45 mm Hg with an aortic valve area index of 0.4 (IQR 0.33–0.57) cm^2^/m^2^ (Table 1). A subgroup of 26 (27.6%) patients met criteria of low gradient (LG) AS defined as mean transvalvular aortic gradient <40 mm Hg with aortic valve area <1 cm^2^. At discharge post-TAVR, the residual mean transvalvular aortic gradient was 10.5 ± 3.8 mm Hg (Table 2).

**FIGURE 2:**
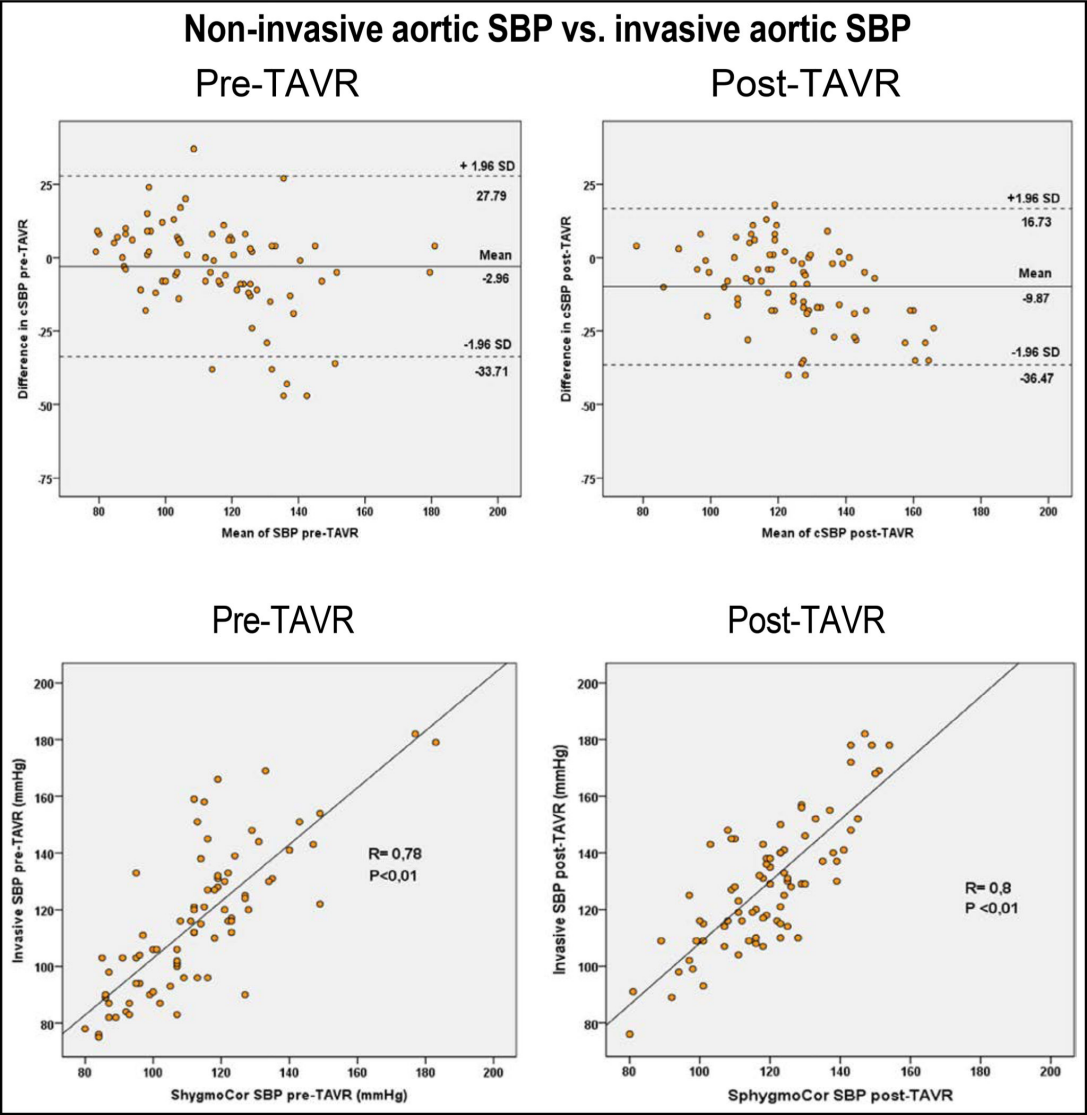
Bland-Altman plots of agreement and Pearson correlation between invasive and non-invasive measurements for aortic systolic blood pressure (SBP) pre-TAVR and post-TAVR

The Bland-Altman plots of agreement between invasive and non-invasive data for central aortic SBP pre- and post-TAVR and the correlation are presented in Figure 2. The mean difference increased after TAVR (from −3 to −9 mm Hg) due to a higher value for invasive SBP measurements, however, the variability was similarly large, ±15 and ±13 mm Hg and Pearson *r* values .78 and .8, respectively.

**FIGURE 3:**
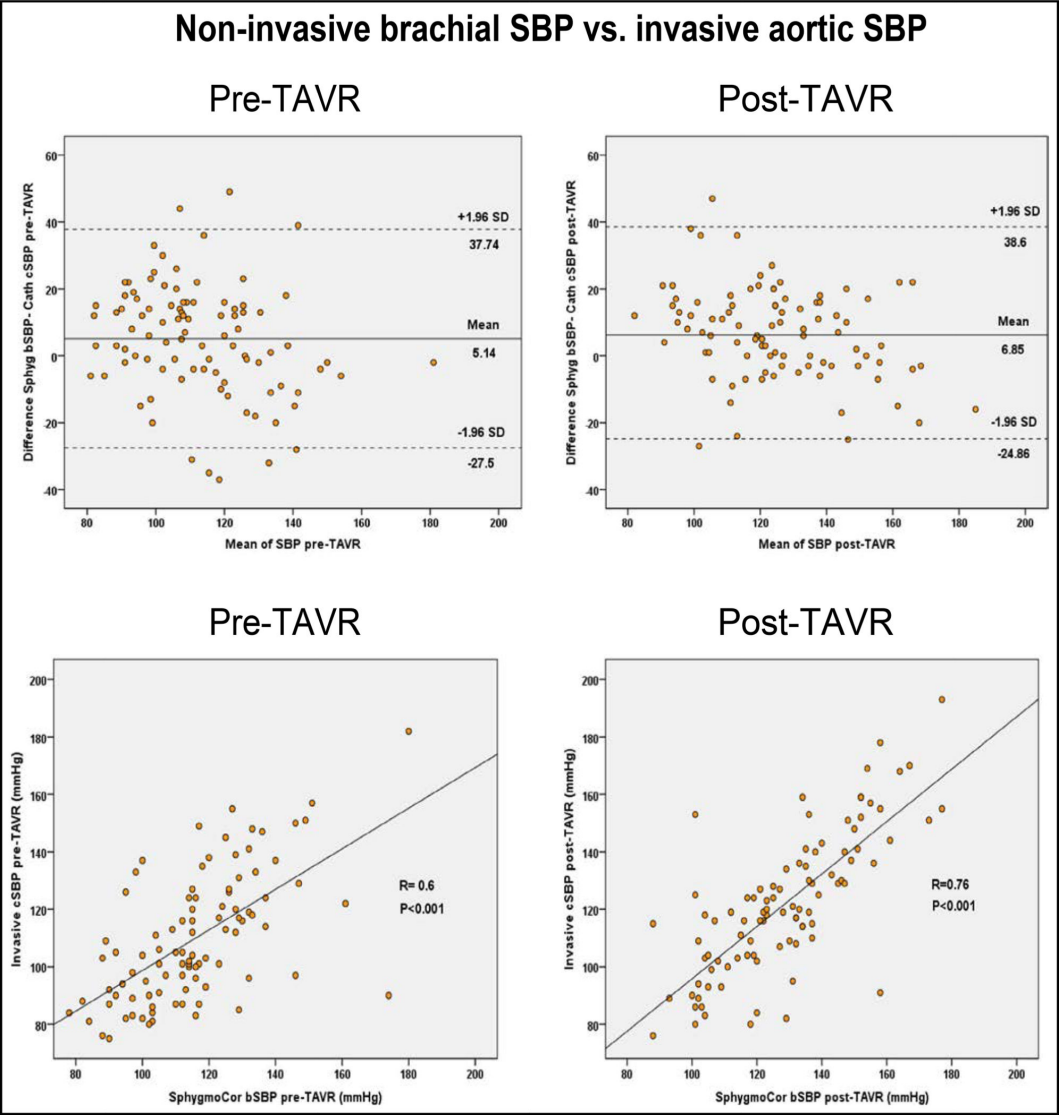
Bland-Altman plots of agreement and Pearson correlation between invasive aortic systolic blood pressure (SBP) and cuff- brachial systolic blood pressure (SBP) measurements pre-TAVR and post-TAVR

The Bland-Altman plots of agreement and the correlation analysis between invasive central aortic SBP and cuff-brachial SBP are shown in Figure 3. The mean differences were positive before and after TAVR (+5 and +7 mm Hg) and the variability was large (±16 mm Hg) in both stages with Pearson *r* values .6 and .76, respectively.

**FIGURE 4:**
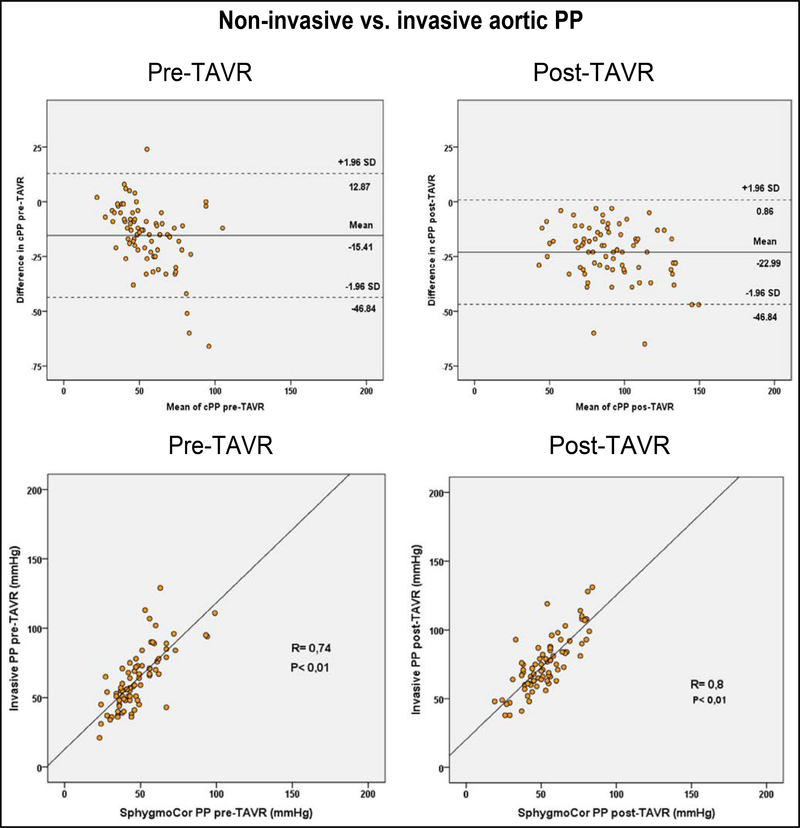
Bland-Altman plots of agreement and Pearson correlation between invasive and non-invasive measurements of aortic pulse pressure (PP) pre-TAVR and post-TAVR

The Bland-Altman plots of agreement and correlation between invasive and non-invasive measurements of central aortic PP pre- and post-TAVR, are shown in Figure 4. Larger mean differences in favor of the invasive measurements were reported both pre- and post-TAVR.

**FIGURE 5:**
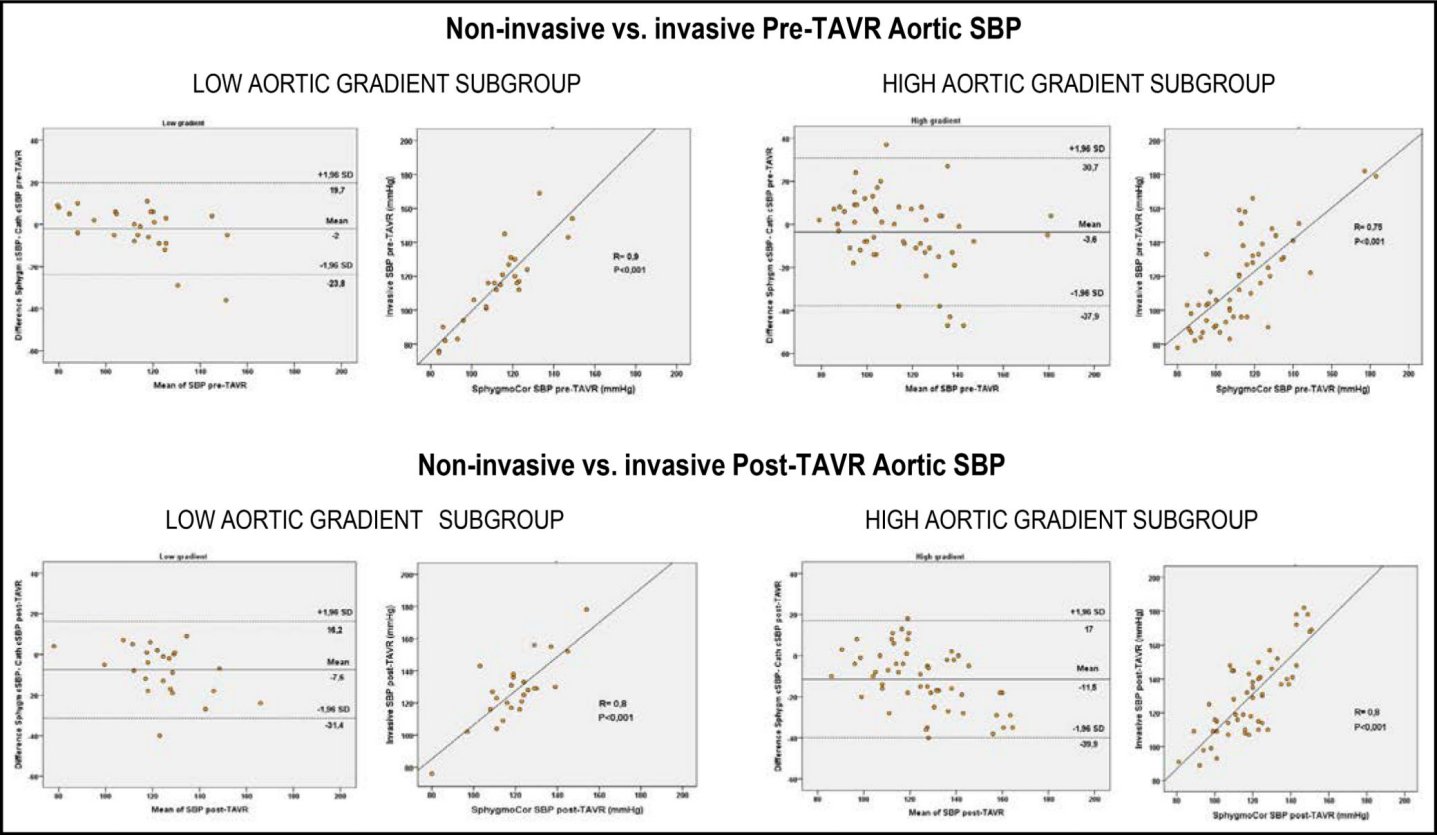
Bland-Altman plots of agreement and Pearson correlation between invasive and non-invasive measurements for aortic systolic blood pressure (SBP) pre-TAVR and post-TAVR in patients with and without low gradient aortic stenosis (mean transvalvular aortic gradient <40 mm Hg)

We conducted an agreement analysis considering different aortic gradient subgroups (Figure 5). Before TAVR a trend for a better, yet suboptimal, agreement was observed in patients with LG aortic as compared with those with high transvalvular gradients (−2 ± 11 vs. −4 ± 17 mm Hg, *p* =.3) with Pearson *r* values .9 and .75, respectively.

**FIGURE 6:**
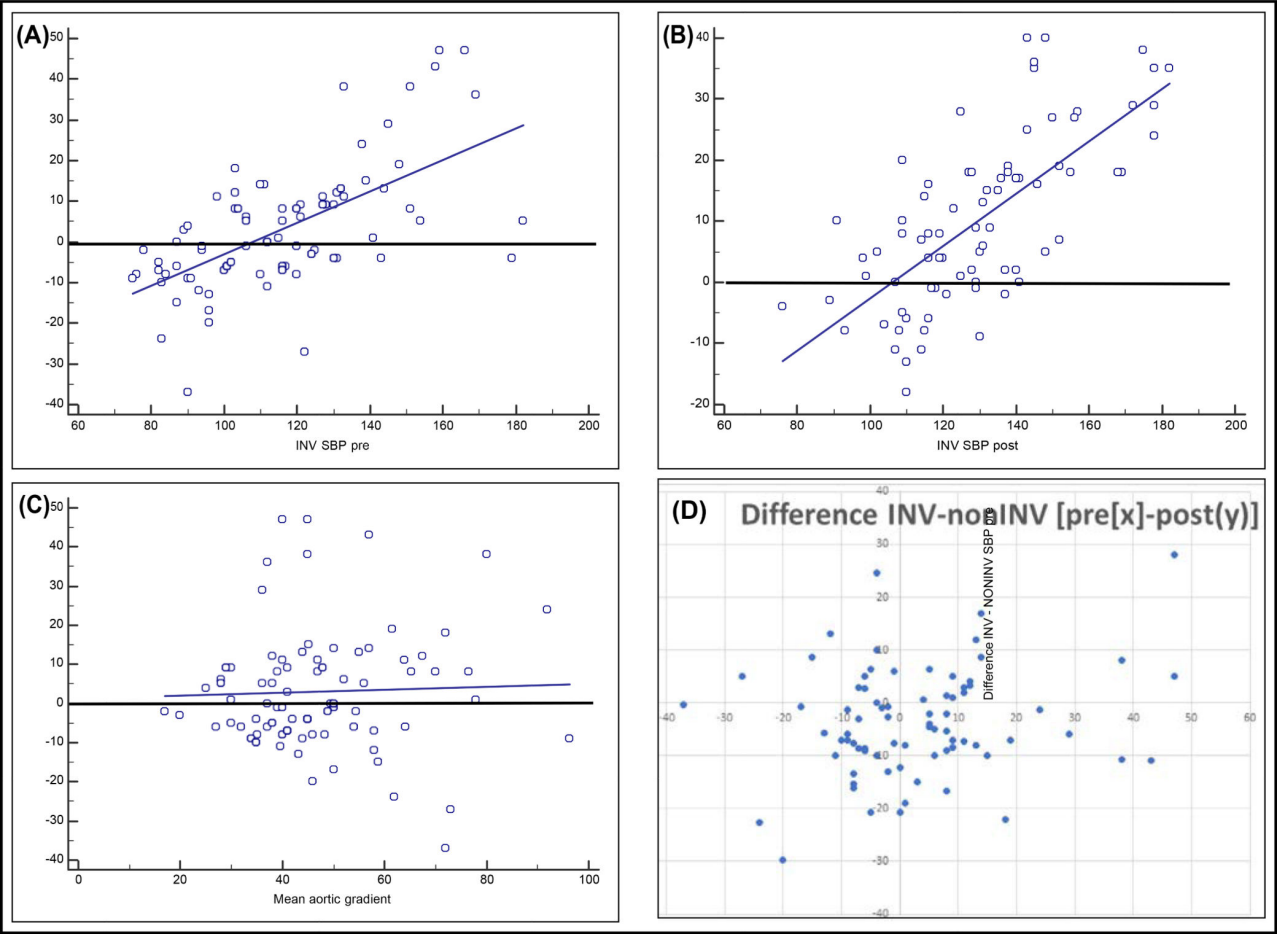
Scatter diagram and regression lines showing graphically the relation between the magnitude of bias (difference of invasive and non-invasive aortic SBP) with invasive aortic SBP before TAVR (A), after TAVR (B), and with the mean aortic gradient before TAVR (C). (D) Difference between invasive and non-invasive aortic SBP, both pre-TAVR (on *X*-axis) and post-TAVR (on *Y*-axis)

The only independent predictor for the magnitude of bias (the difference between invasive and non-invasive aortic SBP) was the value of invasive aortic SBP. In Figure 6A,B is illustrated the relation between this difference and the value of the invasive aortic SBP, both before and after TAVR. The bias was increased when the invasive aortic SBP was over 140 mm Hg. A mean aortic gradient over 40 mm

Hg was associated with larger differences (Figure 6C). The differences between invasive and non-invasive aortic SBP both before and after TAVR are presented over a XY cross diagram in Figure 6D.

## 4 | DISCUSSION

The vascular system is an integral part in the pathophysiology of AS. Evidence gradually accumulates to establish the clinical importance of evaluating vascular properties in guiding the appropriate treatment before and after aortic valve replacement.^5,6^ As this paradigm grows the importance of using the correct and valid tools to evaluate vascular function is of utmost importance. To this end, we have performed the current study aiming to evaluate the validity of measuring non-invasive central blood pressure in patients with AS using the SphygmoCor XCEL^®^ device. We specifically compared the non-invasive data with invasive pressure measurements both before and after valve replacement and in different subsets of AS patients.

The main findings of our study could be summarized as follows:

(1) The agreement between invasive and non-invasive SphygmoCor XCEL measurement for central aortic SBP was insufficient to meet the ARTERY consensus criteria.^8^ (2) Bland-Altman analysis shows lower bias before and a larger bias after TAVR due to a more increased invasive SBP values. (3) The accuracy could be better for patients with low gradient AS and for those with invasive central SBP < 140 mm Hg, so the higher the invasive aortic SBP the larger the bias between techniques. (4) The cuff-brachial systolic BP provided numerically lower degree of agreement and weaker correlation with invasive measurements than the SphygmoCor XCEL measurement for central aortic SBP.

Three previous studies compared the SphygmoCor XCEL device with invasive central blood pressure have been conducted. A study by Shoji et al found that XCEL-derived central SBP under- estimated invasive central SBP −4.6 ± 9.9 mm Hg in 36 patients.^13^ Nonetheless, this study did not fulfill the requirements of a validation study according to ARTERY Society protocols and in any case, the differences were outside the pass criteria.^8^ The only appropriately powered and conducted validation studies for the XCEL device are Gotzmann et al.^10^ and Schultz et al.^11^ In the first study, including 502 patients, non-invasive assessment of aortic SBP was performed by both the SphygmoCor XCEL device and the Mobil-O-Graph NG device and was compared with simultaneous invasive pressure measurement (through fluid-filled catheters). The mean systolic bias of SphygmoCor XCEL device to invasively assessed aortic SBP was −5.0 ± 7.7 mm Hg and with Mobil-O-Graph NG device was slightly but significantly larger (−6.0 ± 10.4 mm Hg, *p* =.011). Both devices slightly underestimated systolic pressure but biases were lower with the SphygmoCor device (*p* <.001 each).^10^ The most recent validation study was aimed to determine the accuracy of the SphygmoCor XCEL cuff device for measuring central blood pressure in 296 patients undergoing coronary angiography.^11^ Central SBP was underestimated and with wide variability (−7.7 ± 11.0 mm Hg). In contrast to the previous study, the SphygmoCor XCEL cuff device was found not to meet the accuracy criteria for non-invasive measurement of central SBP of 5 ± 8 mm Hg.^8^

It is well-known that daily used brachial-cuff SBP also under-estimates the true (intra-arterial) brachial SBP but overestimates the true (intraarterial) brachial DBP. ^14^ These errors lead to inaccu- rate calibration of the cuff waveform and a consequent transfer of error (underestimation) to the estimated central SBP. Furthermore, intra-arterial brachial SBP is underestimated to a greater extent by brachial-cuff SBP at higher SBP levels.^14^ The XCEL device overestimates SBP amplification may be because the GTF is constrained in the ability to detect the true interindividual range in the variability of invasive central-to-brachial SBP amplification.^15^ In a recent review on the accuracy of non-invasive measurement techniques (the vast majority based on applanation tonometry) 22 eligible studies were identified, which validated 11 different commercial devices in 808 study participants. ^16^ The mean error in the estimation of central SBP was of −4.5 mm Hg. As a potential advantage, the SphygmoCor XCEL automated procedure does not necessitate an intense training of staff anymore and the results are far less observer-dependent than previous tonometric approaches, which required placement of the tip of a hand-held high-fidelity tonometer on the patient’s artery.

With regards to patients with the diagnosis of AS, there is only one study previously published aimed to determine the relationship between non-invasively measured brachial pressure and invasively measured central aortic pressure. ^12^ A radial artery tonometry was performed using a SphygmoCor system. This was calibrated from brachial artery blood pressure measured by an oscillometric device.

Transfer function estimates of central systolic pressure obtained from the radial waveform calibrated from brachial pressure were not very accurate (mean difference ± SD, −8 ± 7 mm Hg). The brachial SBP provided a better approximation of the ascending aortic systolic pressure (mean difference ± SD, 2 ± 9 mm Hg) than that derived from a transfer function using either non-invasive or invasive calibration. As relevant limitations in this study stand out the low number of patients (*n* = 14), the mix of cases with moderate and severe stenosis, and the lack of evaluation after valve replacement, Moreover the invasive pressure catheter was placed as close as possible to the aortic valve not considering the pressure recovery phenomena, and thus overestimating the true central aortic pressure.

One of the explanations for these findings was the reduced rate of rise of pressure in AS leading to a reduction in the high-frequency content of the central pressure waveform. It is the high-frequency components of the waveform that are distorted on transmission along the upper limb.^17^ The brachial systolic pressure was only partially approximated to central blood pressure in a number of cases and it is possible that this phenomenon was in part related to early wave reflection in some patients and not in others.

In a recently published study, 20 elderly patients with AS underwent a simultaneous applanation tonometry/CMR protocol for quantification of valvulo-arterial load. CMR provided left ventricular volume and aortic flow simultaneously with radial applanation tonometry pressure acquisition. Derived aortic pressure correlated well with invasive data, like in our study, but agreement data are not shown for central pressure in the manuscript. ^18^

In our study, we observed the underestimation of aortic SBP by the non-invasive method, being this tendency more pronounced after TAVR when SBP is higher (as previously noted). The mean difference was lower (−3 mm Hg) but dispersion was somehow larger than in general population.^10,11^ The non-invasive metrics correlated well with the invasive ones, but correlation is not the intended statistic to consider when aiming a validation study. Having said this, the superior correlation with the central invasive pressure shown for the XCEL estimated central pressure with respect to the cuff-brachial SBP before TAVR would make it the first best candidate to be used in the calculation of non-invasive metrics related to the valvulo-arterial load in patients with AS.

It is not feasible to say that the brachial artery encompasses all the variability seen but rather the outflow from the stenotic valve is a large contributor to this. That all being said a similar amount of variably is seen in the post-TAVR cases which is to suggest that the variability seen is simply what the SphygmoCor XCEL can measure and that in a pre-TAVR or post-TAVR case the variability is similar.

In addition, the population in our study is much older than in previous validation studies, with a higher prevalence of increased aortic stiffness and isolated systolic hypertension, conditions that reduce the accuracy of the non-invasive BP measurement.^19^ Furthermore, AS could induce changes in aortic-vascular system affecting the validity of the GTF, which are not immediately reversed after TAVR. Thus, we cannot exclude the possibility of an improvement in the ac- curacy of the GTF method at longer follow-up after the intervention.

Interestingly the accuracy tended to improve in the subgroup of patients showing a low gradient AS, but only at baseline, may be because of the associated lower values of SBP (when agreement appears to improve) and a relative lower severity of AS. The bias increased after TAVR, most probably due to the increased values of invasive SBP.

### 4.1 | Limitations

The use of fluid-filled catheters to record invasive central and brachial BP, if handled incorrectly, could lead to inaccurate measurement of blood pressure. Nonetheless, a standardized and methodical protocol for the measurement of invasive central BP was implemented, including removal of bubbles from the arterial line, regular flushing, placement of catheter ~5 cm above the valve, and confirming the dynamic response to be within the required range. Undoubtedly, having a simultaneous invasive assessment of brachial pressure would have been helpful in assessing the variability. However, at the time of study design, it was not considered adequate to add more invasive maneuvers in these very elderly patients undergoing TAVR since any intravascular manipulation of catheters can generate complications and even more so in this type of patients.

## 5 | CONCLUSIONS

In patients with severe AS, the agreement of SphygmoCor XCEL with invasive measurement for aortic SBP did not pass the ARTERY Society protocol requirements either before or after TAVR despite showing better correlation than the cuff-brachial pressure. The mean difference between invasive and non-invasive measurements increased along with the invasive aortic SBP and the aortic gradient.

## Figures and Tables

**TABLE 1:** Baseline characteristics

	*N* = 94
Age (years)	81.04 ± 6.7
Female	44 (46.8)
Hypertension	78 (83)
Diabetes	26 (28)
Body mass index	27.8 ± 5.2
Previous myocardial infarction	7 (7.4)
Previous PCI	11 (11.7)
COPD	23 (24.5)
Previous stroke	0
Liver disease	3 (3.2)
Peripheral vascular disease	6 (6.3)
Atrial fibrillation	36 (38.3)
Glomerular filtration rate (ml/min/m^2^)	63.3 ± 18.6
Prior cardiac surgery	7 (7.4)
NYHA class	
I	5 (5.3)
II	45 (47.9)
III	36 (38.3)
IV	8 (8.5)
Previous angina	13 (13.8)
EuroSCORE II	2 (1.5–3.4)
STS score mortality	3.9 (2.3–6)
Baseline echocardiographic data	
Maximal aortic gradient	78.2 (67–92)
Mean aortic gradient (mm Hg)	45 (38–56)
AVA index (cm^2^/m^2^)	0.4 (0.33–0.57)
SV index (ml/m^2^)	46.8 (38.7–53.6)
LVEF (%)	60 (55–65)

*Note:* Values presented as mean ± SD, median (IQ range), or *n* (%).

Abbreviations: AVA, Aortic valve area; COPD, Chronic obstructive pulmonary disease; LVEF, Left ventricular ejection fraction; NYHA, New York heart association; PCI, Percutaneous coronary intervention; SV, Stroke volume.

**TABLE 2:** Procedural and post-procedural data

	*N* = 94
Transfemoral approach	94 (100)
Balloon expandable TAVR	94 (100)
Echocardiographic data at discharge	
Mean gradient (mm Hg)	10.5 ± 3.8
AVA index (cm^2^/m^2^)	1.1 ± 2.7
LVEF (%)	60 (55–60)
Paravalvular aortic regurgitation ≥II	3 (3.2)
In-hospital complications	
Myocardial infarction	1 (1.1)
Stroke or TIA	1 (1.1)
Minor vascular complication (VARC-2)	4 (4.2)
Major vascular complication (VARC-2)	0
Bleeding complication (BARC ≤ II)	9 (9.5)
Bleeding complication (BARC > II)	0
New-onset atrial fibrillation	1 (1.1)
New definitive pacemaker	8 (8.5)
Death	0

*Note:* Values presented as mean ± SD, median (IQ range) or *n* (%).

Vascular complications are categorized according to the Valve Academic Research Consortium (VARC)-2. Bleeding complications are defined according to the Bleeding Academic Research Consortium (BARC).

Abbreviations: AVA, Aortic valve area; LVEF, Left ventricular ejection fraction; TAVR, Transcatheter aortic valve replacement; TIA, Transient ischemic attack.
